# Testing New Concepts for Crop Cultivation in Space: Effects of Rooting Volume and Nitrogen Availability

**DOI:** 10.3390/life8040045

**Published:** 2018-10-06

**Authors:** Silje A. Wolff, Carolina F. Palma, Leo Marcelis, Ann-Iren Kittang Jost, Sander H. van Delden

**Affiliations:** 1 Centre for Interdisciplinary Research in Space (CIRiS), NTNU Samfunnsforskning AS, N-7491 Trondheim, Norway; a.i.kittang.jost@ciris.no; 2 Horticulture and Product Physiology, Wageningen University, PO Box 16, 6700AA Wageningen, Netherlands; cffpalma@gmail.com (C.F.P.); leo.marcelis@wur.nl (L.M.); sander.vandelden@wur.nl (S.H.v.D.)

**Keywords:** life support, hydroponics, transpiration, human space flight, lettuce, greenhouse, conductivity, gas exchange

## Abstract

Long term human missions to the Moon and Mars, rely on life support systems for food production and regeneration of resources. In the EU H2020 TIME SCALE-project, an advanced life support system concept was developed to facilitate plant research and technology demonstration under different gravity conditions. Ground experiments assessed irrigation systems and effects of rooting- and nutrient solution volume. The maximal allowed volume for existing International Space Station research facilities (3.4 L) was able to support cultivation of two lettuce heads for at least 24 days. A smaller rooting volume (0.6 L) increased root biomass after 24 days, but induced a 5% reduction in total biomass at day 35. Regulating effects of nitrate supply on plant water fluxes in light and dark were also investigated. At low concentrations of nitrate in the nutrient solution, both transpiration and stomatal conductance increased rapidly with increasing nitrate concentration. During day-time this increase levelled off at high concentrations, while during nigh-time there was a distinct decline at supra optimal concentrations. Plants supplied with nitrate concentrations as low as 1.25 mM did not show visible signs of nutrient stress or growth reduction. These findings hold promise for both reducing the environmental impact of terrestrial horticulture and avoiding nutrient stress in small scale closed cultivation systems for space.

## 1. Introduction

Since the year 2000, humans have been continuously present in space on the International Space Station (ISS). Due to its relative proximity to Earth and regular space flights, replenishment of resources such as food and water is ensured to the crew onboard ISS. However, future plans for long term human spaceflight beyond the low Earth orbit or establishment of colonies with a larger crew, will bring critical challenges connected to resupply and waste management [1]. To reduce the need for replenishment, transport mass and costs, different concepts for Bioregenerative Life Support System (BLSS) are developed for future in situ food production in space. Higher plants are foreseen to be an essential part of such systems [2,3,4]. As reviewed by Wheeler [5], ground demonstrations and plant research for BLSS have been performed by the major governmental space agencies for the past half century. In addition to ground-based research, crop cultivation experiments under space conditions with reduced gravity are required [1,2,3,6]. Reduced gravity is expected to influence plant physiology, nutrient uptake and thereby growth speed and potentially nutritional value in space grown crops [3,7]. On the ISS, scientific work and technology demonstrations can be performed with fractional gravities including microgravity and simulated Moon and Mars gravity using research facilities with centrifuges [8,9].

**Figure 1:**
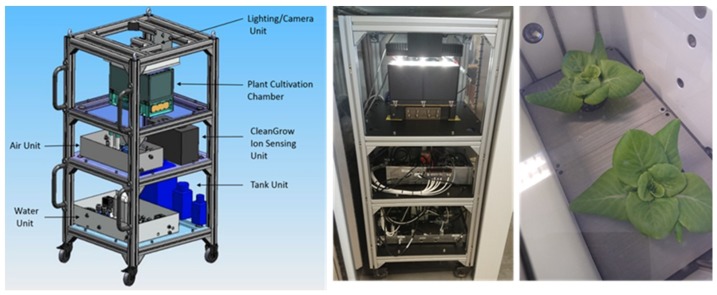
Crop cultivation system prototype developed in the EU H-2020 project TIME SCALE. Credit: DTM technologies and NTNU Social research.

**Figure A1:**
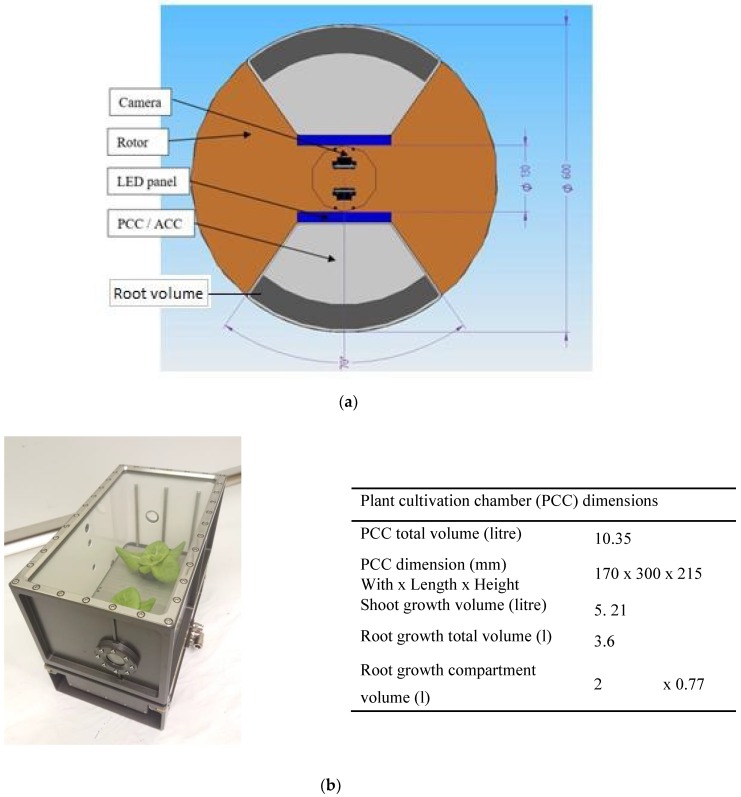
Dimensions of: (**a**) Space centrifuge rotor impression for future space hardware development by DTM technologies and (**b**) plant cultivation chamber (PCC) developed by CMR Prototech for ground testing.

In the EU Horizon 2020 TIME SCALE project, an advanced crop cultivation system prototype was developed for imminent use on an existing centrifuge on the ISS (Figure 1). The crop cultivation concept developed comprises a system to facilitate both technology demonstration and research on algae or plants in fractional gravity. Cultivation can be performed with or without substrate; i.e., deep water culture, which allows pure nutrient research unaffected by soil properties. The system contains two growth chambers per centrifuge rotor with nutrient solution volumes as large as allowed by the centrifuge diameter of 600 mm (Figure A1). Each growth chamber is connected to independent systems that can monitor plant health, provide light, recycle water, and manage nutrient solution electric conductivity (EC) and pH. A multi-ion (NO_3_^−^, H_2_PO_4_^−^, Cl^−^, NH_4_^+^, Mg^2+^, Ca^2+^, K^+^, Na^+^) sensor monitoring system (CleanGrow, Wolverhampton, United Kingdom) is able to accurately detect dynamics in macro nutrient uptake. The crop cultivation chambers are interchangeable with algae cultivation chambers [10,11]. All other parameters required for optimal cultivation (temperature, humidity, and CO_2_) are ensured by integrating the system in an incubator on ground or on the ISS.

Restricted growth volumes, as used in our crop cultivation system (Figure A1), can result in a wide range of physiological and morphological responses [12]. This effect is undesirable because it will confound the fractional gravity effects assessed on the ISS. Moreover, nutrient supply is an important environmental signal that strongly affects root development [13] and shoot gas exchange rates [14,15,16,17]. As gas exchange in micro gravity is severely hindered by the lack of buoyant thermal convection [18], any potential regulatory effects of nutrient concentration on plant water fluxes could be used to enhance transpiration in space crop cultivation systems. The effects of transpiration on nutrient acquisition are well documented (reviewed by Tibbitts [19]): transpiration drives the mass flow of nutrients from the soil to the roots [14], and aids in translocation of nutrients within the plant [20,21]. In turn, nutrient availability can influence transpiration [14,15,16,17]. Although the regulating mechanisms are not clear, nitrogen (N) is among the elements proposed to have a role in regulating plant water fluxes [14,15,16,22,23,24,25]. Plant fertilization with low or restricted N, causing N limitation but not deficiency, has induced increased stomatal conductance and transpiration in maize [16] and bean [15] as compared to deficient and supra optimal concentrations. According to Wilkinson, Bacon and Davies [16] the observed response is stronger in well-watered plants, pointing in the direction of interaction between N concentration and stomatal conductivity.

The current paper aims to address three aspects of plant (*Lactuca sativa*) production in confined and closed loop cultivation systems:

(i) The effect of a restricted rooting volume was studied by comparing a small (0.6 L) and a large (3.5 L) root container. We hypothesize that as longs as the conditions in both containers are similar there will be no effect of the root container.
(ii) The effects of a limited amount of nutrient solution were tested by comparing a 3.4 L nutrient solution for the cultivation of two lettuce heads to plants which receive an unlimited supply of fresh nutrient solution. We hypothesize that plants in both systems will be similar.
(iii) The effects of nitrate concentration on stomatal conductance, transpiration and nitrate uptake in intact lettuce was studied by growing plants on different nitrate concentrations; causing growth limitation but no morphological deficiency symptoms. To look for variations throughout the diel cycle, conductance and transpiration was measured during both dark and light conditions. We hypothesize that nitrate concentration has a regulating effect on plant water fluxes and that the relation between nitrate concentration and transpiration can be represented by a “bell curve” as described by Wilkinson, Bacon and Davies [16]. That is, when nitrate is supplied in a concentration range between 0 and 30 mM plant responses will gradually increase until reaching an “optimum concentration” at which transpiration peaks and then declines as nitrate concentrations becomes supra optimal.

## 2. Materials and Methods

**Figure A2:**
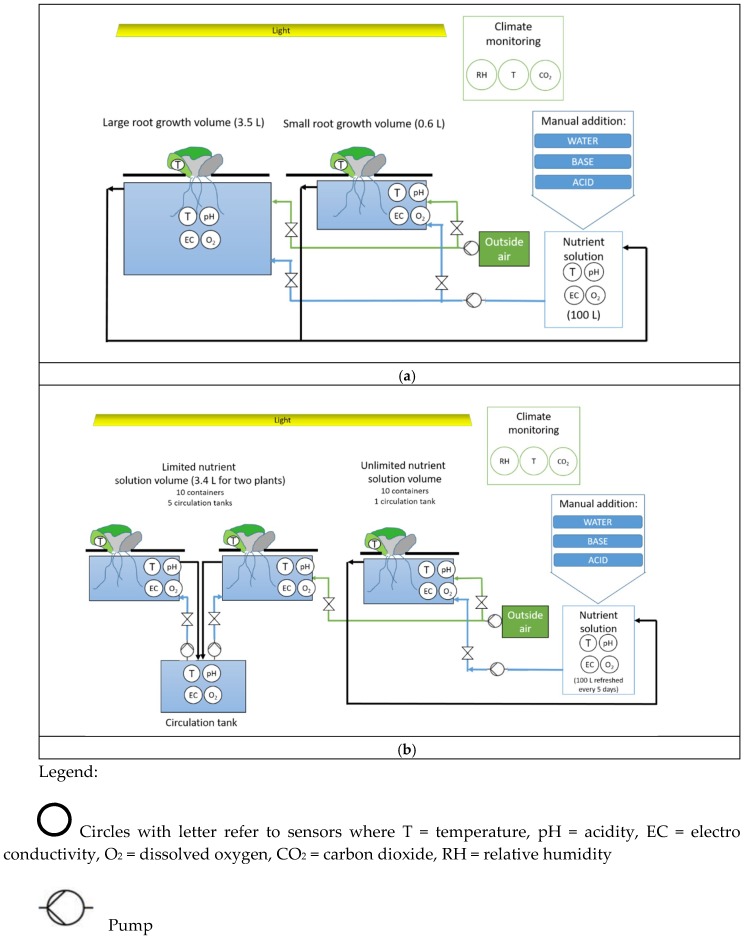
Schematic representation of (**a**) root and (**b**) nutrient volume experiment.

Three experiments were performed: the first two experiments determined the effect of restricted rooting- and nutrient solution volumes (Figure A2), and based on this a third experiment was performed to assess plant responses to various nitrate nutrient solution concentrations.

### 2.1. Plant Material and Growth Conditions

Lettuce (*Lactuca sativa*, cv. Cecilia RZ butterhead) seeds, from Rijk Zwaan Nederland B.V. De Lier, The Netherlands, were sown in round seed holders filled with vermiculite and water. All plants were cultured in climate chambers at Wageningen University under a photoperiod of 16 h. CO_2_ concentration, temperature, and relative humidity (RH) were controlled and recorded by a “Hoogendoorn^®^ climate control system”. Temperature was set to 24/19 °C (day/night), relative humidity to 75% and CO_2_ concentration to 400 ppm (ambient). Light was provided by fluorescent-tubes (T5-36W, Philips, Eindhoven, The Netherlands), average light intensity at plant height was 335 µmol·m^−2^·s^−1^ PAR (Photosynthetic Active Radiation). Harvested plants were directly stored in a cooling box and root and shoot fresh weights, leaf area, leaf number and both rooting volume and length were determined within two hours after harvest. To obtain dry weights (biomass), shoots and roots were dried separately at 70 °C until constant weight (max. 4 days). The leaf area (cm^2^·plant^−1^) was measured using a Li-Cor-3100 (Li-Cor Biosciences, Lincoln, NE, USA) and a flatbed scanner for early growth stages. In the first two experiments, to get an impression of relative differences in stress or nutrient shortages the Dualex Scientific^TM^ was used. The Dualex^TM^ provides relative estimates of concentrations of chlorophyll, flavonoids, and anthocyanins (http://www.force-a.com/en/publications). Plant nitrogen content was determined by a LECO element analyzer at the Chemisch Biologisch Laboratorium Bodem (CBLB) lab of the Wageningen University.

### 2.2. The Effects of a Restricted Rooting- and Nutrient Solution Volume

In the first experiment the effects of rooting volume per se were determined and in a second experiment the effects of a limited nutrient solution were determined. For both experiments the nutrient solution had an EC of 1.65 dS·m^−1^ and was composed of the following ions in mmol L^−1^: NO_3_^−^ 9, NH_4_ 1.5, P-H_2_PO_4_^−^ 1.5, K^+^ 5.5, Ca^2+^ 3, Mg^2+^ 1, SO_4_^2−^ 1.5, Cl^−^ 1.5, Si 0.5, and in µmol L^−1^: Fe 28.1, B 47, Cu 1, Zn 6.4, Mn 1.5, Mo 0.7. EC and pH were measured both in the main solution tank and in the growth units. EC was maintained daily by adding small amounts of deionized water when the EC increased due to evapotranspiration and pH was maintained in the range of 5.6–6.0 with citric acid (0.1 M) and K_2_HCO_3_ (0.1 M) added to the main solution tank when necessary.

For the root volume experiment two container types were used: small root containers, inner dimensions 105 × 105 × 75 mm; i.e., similar size as in the space crop cultivation concept, which were filled with 0.6 L nutrient solution; and large root containers, inner dimensions: 265 × 165 × 115, which were filled with 3.5 L nutrient solution. Ten blocks containing one replicate of each small and large root container were distributed over the climate room. The system was setup such that all root containers received the same nutrient solution from a 100 liter nutrient tank. Solution was pumped (‘Eheim Universal’ 1200 L/h, EHEIM GmbH & Co. KG, Germany) via a distribution tube into the root containers. By adjusting the inlet valves of each container, the flow rate of the nutrient solution was set to approximately 21 L h^−1^. The drain tube in container was located at the upper part of the containers. The drain was collected into a main drainage pipe and returned to the main nutrient solution tank by gravity, creating a closed nutrient solution loop (Figure A2). An air pump (28 L min^−1^) pumped air to a main PVC distribution pipe Ø 4 cm from which air out flow to each root container was controlled per container. This insured the equal distribution of air to maintain O_2_ saturation levels in the root zones of all the units. The 100 L nutrient solution tank was refreshed weekly and as a check samples of the solution were taken every 5 days and sent to the lab facilities of Eurofins Agro NL, Wageningen, the Netherlands, for ion concentration analysis. EC, pH and dissolved oxygen where measured with a calibrated Orion Star™ A329 pH/ISE/Conductivity/Dissolved Oxygen sensor. Four plants were harvested after: 10, 15, 20, 25, 30 and 35 days. Fresh and dry mass of both root and shoot, leaf area, root length and root volume were obtained. Dualex^TM^ measurements were performed on both sides of two leaves per plant just before harvest.

In the second experiment on nutrient solution volume the small root containers (0.6 L) were used. Five blocks containing two root containers each were distributed over the climate room. The two containers were either connected to a container with 3.5 L nutrients solution that was not refreshed; i.e., limited nutrient solution treatment, or to a main system with 100 L that was refreshed weekly, ‘unlimited’ nutrient solution treatment. Environmental conditions for both experiments were similar except for CO_2_ concentration that was 1000 ppm in the nutrient solution experiment opposed to 400 ppm in the rooting volume experiment.

For both the first and second experiment treatment means per plant measurements, i.e., root length, root volume, leaf area and both root and shoot dry and fresh mass were compared per time point using ANOVA for a randomized block design using five blocks (‘aov’ function in R version 3.5.1.). Assessment for significant differences between means was done using Fishers Least Significant Difference (LSD) test (*p* ≤ 0.05).

### 2.3. Plant Responses to Various Nitrate Concentrations

Two experimental runs were performed to determine the effects of nitrate (NO_3_) concentrations on lettuce biomass, nitrogen content, stomatal conductance and transpiration. In the first run (hereafter referred to as Run 1), plants grew in five different nutrient solutions with NO_3_ concentrations ranging from 2.5–30 mM (Table 1). Preliminary analysis of Run 1 indicated a curvilinear response to NO_3_ concentration, with a decline in stomatal conductance (g_s_) and transpiration (E) mainly under dark conditions. Based on this, a second run (Run 2) including more replicates and lower NO_3_ concentrations than 2.5 mM, was performed to further explore the plant responses in the lower concentration range and improve data resolution. Following gas exchange measurements on day 12 (Run 1) and day 8 (Run 2), plants were harvested and measured for root length, leaf number, leaf area, fresh biomass of shoots, and roots separately.

#### 2.3.1. Nutrient solution formulation for nitrate treatments

Based on the root volume experiment, the nutrient solution of the breeding phase and used as starting point for the different nitrate treatments was composed of the following ions in mmol L^−1^: NO_3_^−^ 10, P-H_2_PO_4_^−^ 1.5, K^+^ 13.9, Ca^2+^ 7, Mg^2+^ 2.3, SO_4_^2−^ 7, Cl^−^ 7, Si 0.5, and in µmol L^−1 :^ Fe 28.1, B 47, Cu 1, Zn 6.4, Mn 1.5, Mo 0.7. To allow for the various N concentrations while keeping EC constant, EC was kept at 3.3 dS·m^−1^ by substituting NO_3_^−^ with SO_4_^−^ and Cl^−^ (Table A1). At seedling emergence, four days after sowing (DAS) plants were exposed to a 1/3rd (1.1 dS·m^−1^) nutrient solution, six DAS to a 2/3rd (2.2 dS·m^−1^) and eight DAS to a full strength (3.3 dS·m^−1^) nutrient solution. At 16 DAS, plants with similar size and dimension were transferred to the experimental root containers (265 × 165 × 115 mm, same as large root container in the root volume experiment) prefilled with the different nutrient solution treatments (3.5 L in each container, filled to 35mm under the lid). Nutrient solution composition was determined before onset of treatments (without plants) and at experiment end (after harvest). Samples of 50 ml were taken from each growth pot and analyzed for all essential plant nutrients (Eurofins^®^ Netherlands, Wageningen).

#### 2.3.2. Stomatal Conductance and Transpiration Rate Measurements

After exposure to the different nitrate concentration treatments (Table 1), stomatal conductance and transpiration were measured during the light and the dark period with a LI-6400 Portable Photosynthesis System (Li-Cor Biosciences, Lincoln, NE, USA) equipped with a leaf chamber fluorometer (area = 2 cm^2^) and controlled LED source (90% Red + 10% Blue). Three hours after start of the dark or light period five measurements per leaf, one every five seconds were recorded. In order to reduce the variance between plants the distal part of the last fully expanded leaf blade, avoiding the leaf vein, were measured. Rates of night-time transpiration were measured with the same climatic settings as the in the cultivation chamber and a PPFD of 0 µmol m^−2^s^−1^. Low intensity green light (<0.07 µmol m^−2^s^−1^ at plant level) from a fluorescent tube was used as working light during dark measurements. All plants in one block were measured consecutively.

In Run 1, analyses showed an inaccuracy in nutrient solution mixing and all treatments were replaced prolonging the experiment with four days. This affected plant size at the time of measurements and its` potential impact on the results are included in Section 3. Consequently, the plants in Run 1 were measured on day 11 (dark) and 12 (light) after onset of treatments, while in Run 2 plants were measured on day 7 (dark) and 8 (light) after onset of treatments. As suggested by I. Matimati (pers. comm. January 2017), Run 2 included additional measurements 24 h after onset of treatments (in dark only) to look for plant initial responses to nitrate treatments.

#### 2.3.3. Statistical Set-Up and Analysis

Both experiment runs were set up as complete randomized block designs; comprising 5 treatments, one plant per treatment and 6 blocks (replicas) in Run 1, and 7 treatments and 8 blocks in Run 2. To analyze the effects of nitrate concentration on transpiration (E) and stomatal conductance (g_s_), we ran linear mixed effects regression models (LMERs) from the lmer function in package lme4 for *R*. The five data points per leaf sample from the LiCor 6400 photosynthesis system (E and g_s_) were aggregated using the median. LMERs were run with NO_3_ concentration as a continuous variable (log-transformed and adding 0.1 to avoid log of 0). First, we built a global model for model selection based on the Akaike Information Criteria corrected for sample size (AICc; Burnham [26], Table A2). The global model included the main effects of experimental run (two-level factor) and light conditions (two-level factor) and their two-ways interactions with both a linear and a quadratic term for NO_3_. Plant ID nested within Block was included as random intercept effects. In the model selection, all possible subsets of this global model were included, keeping the random structure constant. A similar approach was used to evaluate effects of nitrate concentration on plant biomass and root and shoot tissue N content, except that the factor Light Conditions (and hence Plant ID) was not included as measurements were performed only once at harvest. The residuals were tested for normality and homogeneity of the distribution using Shapiro–Wilk test and QQ-plots. All analyses were performed in R version 3.3.2 (R core Team, 2016).

## 3. Results and Discussion

### 3.1. Root Volume Experiments

**Figure 2:**
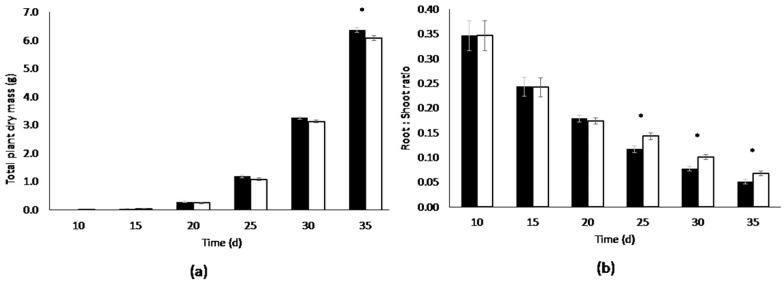
(**a**) Total plant dry mass and (**b**) Root:Shoot ratio per harvest, i.e., time from sowing, of lettuce plants grown in the large 3.5 L (black bars) and small 0.6 L (white bars) containers. Means per time point followed by * differ significantly (*p* < 0.05). Error bars represent the standard error (α < 5%).

Only at final harvest, 35 days after sowing (DAS), shoot dry mass from large (3.5 L) root containers was 10% higher than from the small (0.6 L) root containers (Table A3). Total plant dry mass from the large containers was only 5% higher, reflecting a different Root:Shoot ratio. Root:Shoot ratio of the container types already differed from 25 DAS onwards (Figure 2). The plants in the small root containers accumulated more root biomass than the plants in the large root containers (Table A3). However, before 35 DAS plant characteristics other than root biomass; i.e., plant total dry mass, leaf area, plant water and nutrient content did not differ significantly between container sizes (Table A3).

Daily in situ measurements of root growth conditions: dissolved oxygen content, pH, and electric conductivity (EC) did not reveal significant differences between the root containers. However, it could be that higher root density in the small containers created local micro environments with lower oxygen. Peterson, et al., [27] reported a significant decline in root respiration capacity that correlated with a reduction in root/shoot ratio of tomato plants that were grown in small root containers in a flow through hydroponic system. But the study of Peterson, et al., [27] was done on tomato while lettuce is known to generally handle reduced O_2_ very well, with a minimum dissolved oxygen concentration for proper root respiration of 78 µmol·L^−1^ (30% saturation at 25 °C) [28] and at least 125 µmol·L^−1^ is recommended for optimum lettuce growth [29]; i.e., approximately 50% saturation at 25 ˚C. Due to constant aeration and nutrient solution circulation, the measured dissolved oxygen in our cultivation system was always close to 100% O_2_ saturation (>250 µmol·L^−1^) and the nutrient solution was stable. However, Poorter, et al., [30] reported, that even in the absence of symptoms of nutritional imbalance, restricted hydroponically grown plants do show a decrease in photosynthesis and growth. Although the reasons for this observation are unknown, it could be that the higher root density resulted in locally lower nutrient concentrations in the inner parts of the root system. The fresh nutrient solution was pumped in at the bottom of the container and most likely followed the path of least resistance around the developing rooting system; thereby creating a relatively high local nutrient concentration at the outside of the roots resulting in a more proliferated root system [31,32]. This response is also observed in case of plant to plant competition [32].

Another explanation in the direction of a competition response is that local root exudate concentration (unmeasured) might have been higher in the small root compartment because of higher root density, therewith triggering a competition response of increased root growth to capture nutrient resources.

Aside from signals in form of root exudates or nutrient concentration gradients, roots can respond to physical touching per se [33]. The boundaries of the limited growth environment could, therefore, in itself have caused the alternation in root shoot ratio and a more proliferated rooting system.

Currently there is no literature found that indicates the minimal or optimal root volume for hydroponic water culture systems. Given that eventually at final harvest (35 DAS) shoot fresh mass in the small root container was 10% lower than in the large container, an important practical message for the growers that push toward an ever decreasing rooting volume in vertical farms is that a small root compartment can reduce yields, regardless of optimal supply of water, nutrients and oxygen.

### 3.2. Nutrient Solution Volume Experiments

In order to determine the effects of mass constraint of the nutrient solution on the rotor, plants growing on a relatively unrestricted amount of nutrient solution (100 L for 10 plants refreshed weekly) were compared to those growing on a restricted volume of 3.4 L for 2 plants while the nutrient solution was not refreshed during the cultivation period of 24 days. The different solution volumes did not significantly affect plant characteristics such as plant shoot or root biomass, root length, root volume, leaf area, plant water and nutrient content, plant morphology and chlorophyll and flavonoid content (summarized in supplementary material S1). During the first three weeks the electric conductivity (EC) in the restricted growth volume declined very slowly (0.014 dS·m^−1^·d^−1^); after around 22 days the EC started to decline slightly faster (0.066 dS·m^−1^·d^−1^) (Figure S2). Nutrient solution analysis showed that during the first 22 days NH_4_^+^ and NO_3_^−^ in the root volume were lowered in equal rates of around 0.058 mmol·L^−1^·d^−1^. This suggests a NH_4_^+^ and NO_3_^−^ consumption of approximately 0.099 mmol·d^−1^·plant^−1^. Towards harvest from day 20 onwards, coinciding rapid biomass accumulation per unit of time, NO_3_^−^ removal from the container (Figure S3) abruptly increased to 1.39 mmol·d^−1^·plant^−1^.

This rapid decrease together with the shoot that overgrows the dimensions of the growth volume (Figure S4) and the change in biomass distribution of root and shoot (Figure 2) is the reason for choosing a maximal experimental time for this crop cultivation system of 24 days. That is, provided that growth conditions are set to 24/19 °C (day/night), 75% RH, 335 µmol·m^−2^·s^−1^ PAR, and 400 ppm CO_2_. Note that nutrient consumption rate in this experiment was relatively high as plants were growing at 1000 ppm CO_2_ opposed to 400 ppm used in the other experiments; causing biomass accumulation to be almost twice the amount at harvest; i.e., 2.5 g versus +/- 1.3 g for all other experiments in this paper.

### 3.3. Nitrate Concentration Experiments

#### 3.3.1. Effects on Stomatal Conductance and Transpiration

**Figure 3:**
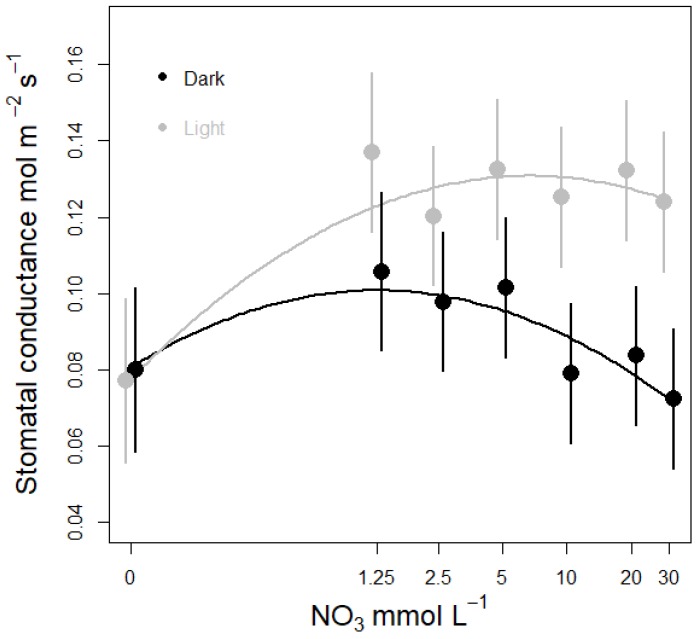
Effect of nitrate level on stomatal conductance in lettuce plants supplied with different nitrate concentrations for one week. Stomatal conductance was measured under light (grey curve) and dark (black curve) conditions. Circles and error bars represent mean (n = 14) ± SE from a model replacing the continuous NO_3_ concentration (i.e., as covariate) with NO_3_ concentration as factor, and Experimental Run as random factor.

**Figure 4:**
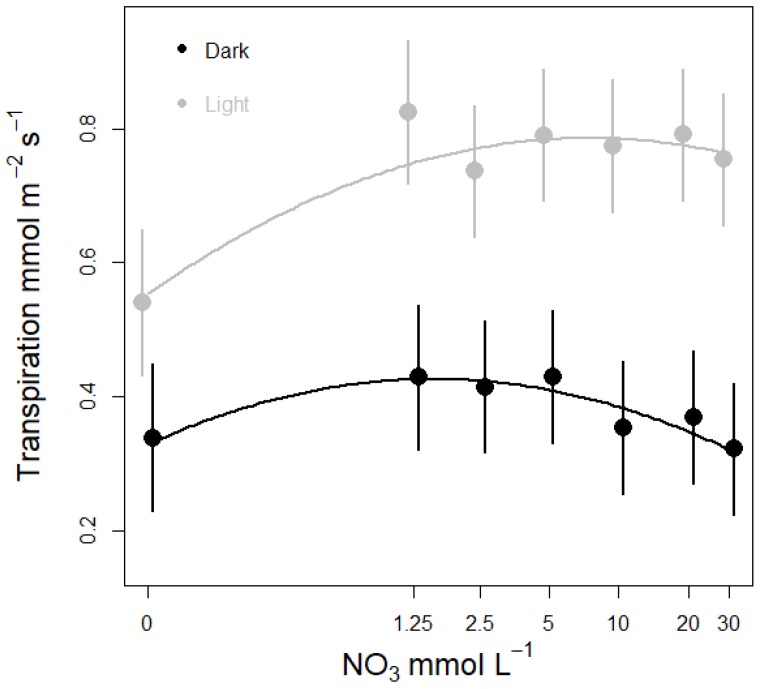
Effect of nitrate concentration of the nutrient solution on leaf transpiration in lettuce plants supplied with different N concentrations for one week. Leaf transpiration was measured under light (grey curve) and dark (black curve) conditions. Circles and error bars represent mean (n = 14) ±SE from a model replacing the continuous NO_3_ concentration (i.e., as covariate) with NO_3_ concentration as factor, and Experimental Run as random factor.

**Figure A3:**
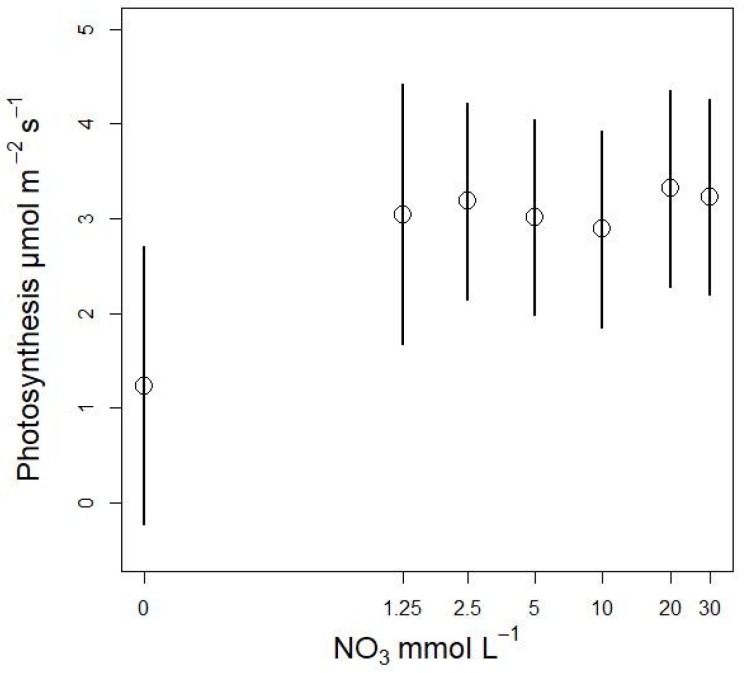
Effect of nitrate concentration of the nutrient solution on photosynthesis in lettuce plants supplied with different N concentrations for one week. Data shown represent measurements performed during light conditions. Circles and error bars represent mean (*n* = 14) ± SE from a model replacing the continuous NO_3_ concentration (i.e., as covariate) with NO_3_ concentration as factor, and Experimental Run as random factor.

Nitrate concentration affected stomatal conductance (g_s_) and leaf transpiration (E) in lettuce plants grown in water culture (Table 2, Figure 3 and Figure 4). The response was similar across Run 1 and 2, but showed a different response under light and dark conditions (Table 2, Figure 3 and Figure 4). During the light period, both stomatal conductance and leaf transpiration increased rapidly when moving from 0 to 1.25 mM NO_3_, and then showed almost no response at concentrations above ~1.25 mM (Figure 3 and Figure 4). Under dark conditions, g_s_ and E also increased from 0 to 1.25 mM, but less steeply than under light conditions. The highest night time g_s_ and night E was measured among plants supplied with between 1.25 and 5 mM NO_3_, and then declined with increasing NO_3_ concentrations above 5 mM (Figure 3 and Figure 4). Model selection included the 0 function (no effect of N dose), a linear function and the quadratic function, with the quadratic function giving the best fit (Table A2). We run a similar model selection for leaf photosynthesis versus nitrate concentration, and the top ranked model suggested no effect of N dose on photosynthesis. Accordingly, there was only a tendency for increased leaf photosynthesis when increasing nitrate concentration from zero to 1.25 mM (Figure A3).

A suggested role for transpiration in dark is acquisition of nutrients when availability is limited [14,25,34]. The stomata of many species remain partly open during the night, and seem to be regulated in many ways similarly to daytime stomatal conductance [34]. The observed increase in transpiration and conductance among plants supplied with NO_3_ levels between 1.25–5 mM, as well as the decline at higher concentrations, suggest that dark transpiration might play a role in nutrient acquisition. Stomatal closure seems to be more sensitive to nutrient availability during the dark period opposed to the light period. As in the dark stomatal closure is decoupled from photosynthesis and radiation.

For the concentration range 0 to 5 mM the measured responses in g_s_ and E followed a curve similar to responses reported for bean [15] and maize [16]. These authors used growth systems with solid growth media and only measured g_s_ and E under light conditions. However, in bean and maize the response to various N concentrations was stronger than our measured responses in lettuce plants. In addition, g_s_ start to level off at 10 mM in maize while for lettuce it declines only slightly from about 5 mM onwards, and shows a significant response to higher nitrate concentrations in dark only (Figure 3 and Figure 4). These differences could be explained by the nutrient depletion zone forming in the soil rhizosphere interface [14,25], justifying a stronger response and slower down regulation of the response when compared to soilless systems. Among the few reported studies of N concentration effects in soilless culture, Senbayram, et al., [35] also reported increased night time g_s_ among plants supplied with NO_3_ concentrations between 3–5 mM compared to N deficient plants receiving 0–0.4 mM NO_3_. However, more research is required to fully understand the impact of night time regulation of transpiration and stomatal conductance.

To study the immediate NO_3_ response, before plants acclimated to the changes in NO_3_ level, Run 2 included measurements after 24 hours exposure to treatments. After 24 hours there was no effect of nitrate concentration on stomatal conductance or transpiration (data not shown). However, small plant size and high variation between individual lettuce plants could have concealed the responses to the NO_3_ treatments.

#### 3.3.2. Plant Biomass Response to NO_3_ Concentration

**Figure 5:**
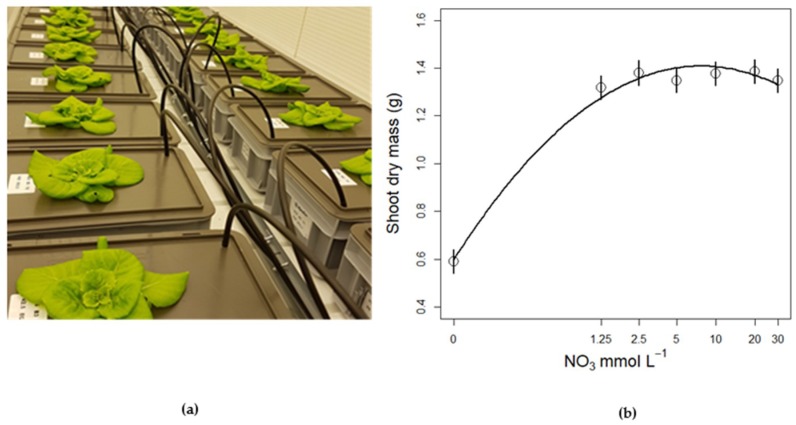
(**a**) Lettuce plants growing in separate growth containers with different nutrient solutions; (**b**) Effect of nitrate level on lettuce shoot dry mass (from Run 2). Circles and error bars represent mean (*n* = 8) ± SE from a model replacing the continuous NO_3_ concentration (i.e., as covariate) with NO_3_ concentration as factor.

**Figure A4:**
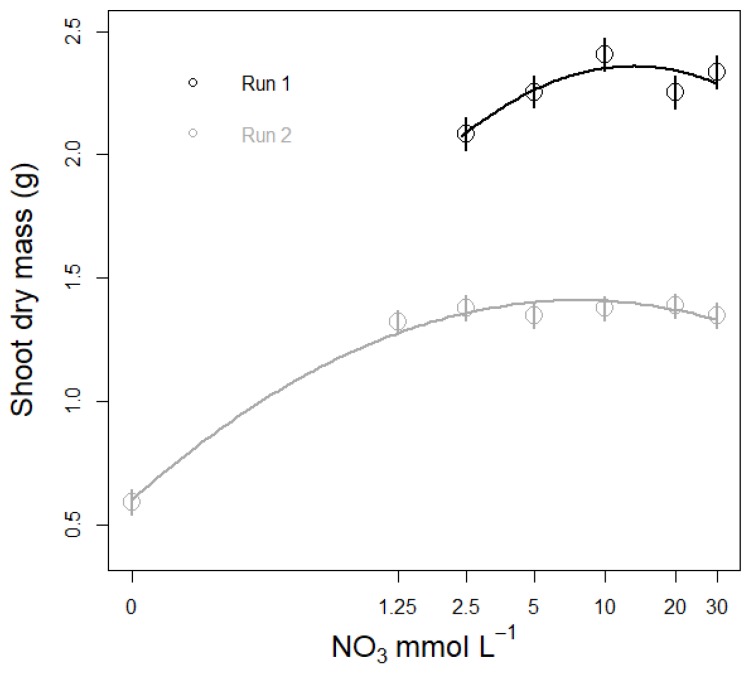
Effect of nitrate level on lettuce shoot dry mass for Experimental Runs 1 (black curve) and 2 (grey curve). Circles and error bars represent parameter estimates and standard errors from a model replacing the continuous NO_3_ concentration (i.e., as covariate) with NO_3_ concentration as factor.

Expectedly, nitrate concentration of the nutrient solution affected the biomass and the plants grown in the zero NO_3_ treatments showed strong growth retardation. There was also a difference in the response between Run 1 and 2, which can be explained by the longer duration of Run 1 resulting in bigger plants (Table A5, Figure A4). Model selection and parameter estimates from the top-ranked linear models for the effects of NO_3_ supply on lettuce plant shoot dry mass are shown in tables A4 and A5, respectively. In Run 2, the additional treatments (0 and 1.25 mM N) showed that shoot dry mass increased strongly when nitrate concentration increased from 0 N to 1.25 mM (Figure 5b), however, higher nitrate concentrations did not seem to affect plant biomass accumulation (Figure 5).

To check for potential confounding effects of individual plant biomass on plant water fluxes, the shoot dry mass was added (in interaction with experiment run) in an initial global model. Including individual plant shoot dry mass in the global model did not change the top ranked model, confirming that the observed responses in plant water fluxes was not due to plant size. Thus, plants supplied with nitrate concentrations as low as 1.25 mM did not show visible signs of nutrient stress or growth reduction. This indicates that growing plants in water culture with roots directly immersed in the nutrient solution allows for lower N concentrations than in systems with solid growth media. Leaf area and leaf number responses were similar to biomass responses (data not shown). For small scale closed cultivation systems for space, where water culture is the preferred system [2,7] and where nutrient solution volumes are limited, this is good news as it makes such systems more robust towards nutrient stress. Also, for terrestrial horticulture this indicates that lowering the nitrate supply and reducing environmental impact could be implemented without yield reduction.

#### 3.3.3. N Amount in Plant Tissue

**Figure 6:**
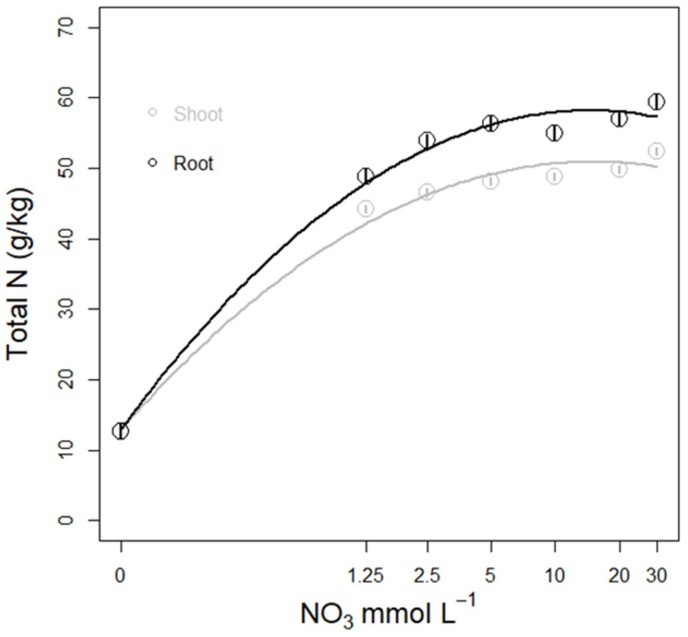
Effects of nutrient solutions with varying NO_3_ levels for one week on N concentration in shoot (grey) and root (black) tissues in lettuce plants (from Run 2).

**Figure A5:**
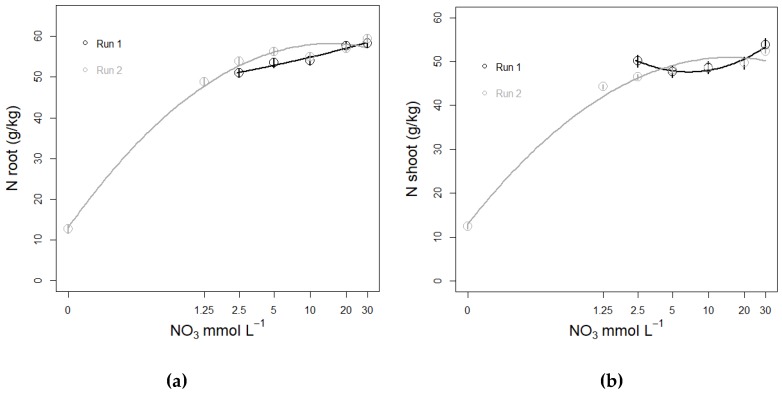
Effects of nutrient solutions with varying NO_3_ levels for one week on N concentration in (**a**) roots and (**b**) shoot tissues in lettuce plants. Circles and error bars represent parameter estimates and standard errors from a model replacing the continuous NO_3_ concentration (i.e., as covariate) with NO_3_ concentration as factor.

The NO_3_ concentration in the nutrient solution affected the nitrogen content (measured as total N) in plant shoots and roots, generating a curvilinear response curve to N concentration in the nutrient solution (Table A6, Figure 6). There was also a difference between Run 1 and 2, with a different slope between the two runs. In Run 1, the initial error in nutrient solution formulation affected the tissue N concentration, making the slope of the curve hard to interpret (Figure A5). In Run 2, both root and shoot N concentration rapidly increased when NO_3_ concentration in the nutrient solution increased from 0 up to 2.5 mM, and then diminished (Figure 6). The N concentrations in tissues seem to level off when supplied with nitrate concentrations above 5 mM, strengthening the proposition that N assimilation in response to nitrate concentration of the nutrient solution is regulated by a decrease in night-time stomatal conductance and transpiration.

### 3.4. Relevance for Future Crop Cultivation in Space

Previous experiments on the International Space Station (ISS) have proven water and nutrient supply to be a challenging aspect of plant growth in space, and illustrated the need for upgraded water and nutrient supply systems [3,8,36]. In TIME SCALE, new concepts for plant cultivation including water culture and nutrient recycling have been developed that can be implemented e.g. in Biolab on the ISS. In addition, the present study adds to the knowledge required for higher plant space cultivation in two ways. Firstly by demonstrating that lettuce can be cultivated without signs of growth retardation or nutrient stress within the volumes allowed on existing rotors. Secondly by adding to the knowledge on NO_3_ regulation of plant water fluxes. Supplying plants with NO_3_ concentrations between 1.25 and 5 mM seems to increase stomatal conductance and transpiration, which would be beneficial under reduced gravity conditions [18]. However, reported responses are highly variable between different species [14], and the number of days a closed and confined cultivation system can support cultivation varies with container size and species. For lettuce in our experimental setup, the maximum time would be approximately 24 days. After 24 days the confounding effects of growth- and nutrient solution volume might impact the gravitational treatment. 

In addition, and with regard to the limitation on nutrient solution replenishment in remote cultivation systems, the ability of lettuce to grow vigorously when supplied with N concentrations as low as 1.25 mM indicates that water culture allows plants to better utilize the nutrients supplied and thus making such systems less susceptible to nutrient stress. 

## 4. Conclusions

For space plant research both the confined environment and mass restriction favor recycling of all resources. Soilless cultivation systems provide effective control in terms of nutrient solution monitoring, adjusting, and recycling. The deep water culture with limited root- (0.6 L) and nutrient solution volume (3.4 L per two plants) used in this study, provided stable and reliable plant growth and high biomass production over a period of at least 24 days. Nitrate concentrations as low as 1.25 mM did not reduce biomass and plant N content.

As expected, the absence of nitrate in the nutrient solution results in low transpiration (E) and conductance (gs). When moving from zero to 1.25 mM the increase in E and g_s_ was much steeper in light than in dark. At concentrations above 1.25 mM, no response was detected in light, while night time g_s_ and night E decreased in response to nitrate concentrations above 5 mM.

## Figures and Tables

**Table 1:** Fertilization treatments with different N concentrations used in the two experimental runs.

	Run 1	Run 2
Nutrient Solution	NO_3_ (mmol L^−1^)	NO_3_ (mmol L^−1^)
1	30	30
2	20	20
3	10	10
4	5	5
5	2.5	2.5
6		1.25
7		0

EC was kept at 3.3 dS·m^−1^ by substituting the NO_3_ with SO_4_ and Cl. pH was set to 5.7. Nutrient solution composition and salt recipes for all treatments are given in appendix x.

**Table 2:** Parameter estimates (β) and standard errors (SE) of fixed effects in the top-ranked linear mixed effects model of lettuce gas exchange responses to solution N concentrations. “Intercept” is the estimate for Run 2, under Dark conditions, and when log (NO_3_ concentration + 0.1) is zero. “Run 1” is the main effect of Run; i.e., the estimated difference from Run 2. “Light” is the main effect of light conditions; i.e., the estimated difference from Dark. The two parameters “NO_3_ concentration” and “NO_3_ concentration ^ 2” give the curvilinear (i.e., quadratic) response under Dark conditions, where “NO_3_ concentration” is the slope when log (NO_3_ concentration + 0.1) is zero, and “NO_3_ concentration ^ 2” is the curvature; i.e., the decrease in slope with increased concentration. “NO_3_ concentration: Light” is the difference in slope from Dark. Standard deviations (SD) and number of groups (n) are given for random effects on the intercept. 85 plants nested in 6 blocks (Run 1) and 8 blocks (Run 2) were measured.

	Conductance (g_s_)		Transpiration (E)	
Fixed effects:	β ± SE	*P*-value	β ± SE	*P*-value
Intercept (i.e., Run 2, Dark)	0.12 ± 0.0078	<0.001	0.5 1± 0.034	<0.001
Run 1	0.032 ± 0.0091	<0.001	0.18 ± 0.040	<0.001
Light	0.019 ± 0.0067	0.005	0.31 ± 0.030	<0.001
NO_3_ concentration	0.0020 ± 0.0033	0.56	0.013 ± 0.014	0.37
NO_3_ concentration ^ 2	−0.0030 ± 0.0011	0.011	−0.012 ± 0.005	0.011
NO_3_ concentration: Light	0.0096 ± 0.0029	0.001	0.038 ± 0.013	0.005
Random effects:	SD	N	SD	n
Plant ID	0.025	85	0.001	85
Block	0.009	14	0.042	14
Residual	0.029		0.134	

## Supplementary Materials

The following are available online at http://www.mdpi.com/2075-1729/8/4/45/s1, Figure S1: Ion concentrations in nutrient solution volume experiment, Figure S2: Effects of nutrient solution volume over time, Figure S3: EC and pH in nutrient solution volume experiment, Figure S4: Rosette growth model

## Appendix A

**Table A1:** Nutrient solution formulation table for the nitrate concentration experiments.

**N-NO₃ Treatment**	P-H₂PO₄	K	Ca	Mg	S-SO₄	Cl	Si
**30.00**	1.50	13.90	7.00	2.30	0.50	0.00	0.50
**20.00**	1.50	13.90	7.00	2.30	3.50	4.00	0.50
**10.00**	1.50	13.90	7.00	2.30	7.00	7.00	0.50
**5.00**	1.50	13.90	7.00	2.30	8.63	8.75	0.50
**2.50**	1.50	13.90	7.00	2.30	9.50	9.50	0.50
**1.25**	1.50	13.90	7.00	2.30	9.88	10.00	0.50
**0.00**	1.50	13.90	7.00	2.30	10.33	10.35	0.50

**Table A2:** Model selection results for the effects of NO_3_ supply on lettuce plant water fluxes, showing the top ranked models for (a) stomatal conductance and (b) transpiration. All models include plant ID and block as random effects on the intercept. Cross indicates that the fixed effect was included in the model.

Model Rank	Run	NO_3_	NO_3_^2	Light	Run:NO_3_	NO_3_:Light	NO_3_^2:Light	AIC	ΔAIC	Loglik
(a) Conductance										
1	+	+	+	+		+		-622.4	0.00	320.76
2	+	+	+	+		+		-620.3	2.08	320.85
3	+	+	+	+	+	+		-620.2	2.19	320.79
4	+	+	+	+		+	+	-620.1	2.25	320.77
5	+	+	+	+	+	+		-618.7	3.70	321.18
(b) Transpiration										
1	+	+	+	+			+	-119.5	0.00	69.32
2	+	+	+	+		+	+	-117.5	2.05	69.42
3	+	+	+	+			+	-117.4	2.14	69.38
4	+	+	+	+	+		+	-117.3	2.18	69.36
5	+	+	+	+	+	+	+	-116.0	3.54	69.82

**Table A3:** Effects of a restricted rooting volume on several plant characteristics. Means are followed by their s.e. (*n* = 5). Means from the small root container (0.6 L) followed by an asterisks (*) are significantly different from the large root container (3.4 L).

Days After Sowing (d)	Container.volume (L)	Root Dry Mass(g) [dry weight]	Shoot Dry Mass(g) [dry weight]	Total Dry Mass (g) [Plant dry weight]	Root:Shoot Ratio	Leaf Area (cm^2^)	Specific Leaf Area (cm^2^·g^−1^)	Biomass (%) ^#^
**10**	**3.5**	0.00218 ± 0.00	0.00670 ± 0.00	0.0089 ± 0.00	0.347 ± 0.01	3.08 ± 0.10	356 ± 10.7	8.30 ± 0.25
	**0.6**	0.00218 ± 0.00	0.00670 ± 0.00	0.0089 ± 0.00	0.347 ± 0.01	3.08 ± 0.10	356 ± 10.7	8.30 ± 0.25
**15**	**3.5**	0.00935 ± 0.00	0.0393 ± 0.00	0.0486 ± 0.00	0.243 ± 0.02	18.1 ± 0.27	373 ± 8.23	6.48 ± 0.15
	**0.6**	0.00868 ± 0.00	0.0366 ± 0.00	0.0453 ± 0.00	0.242 ± 0.01	17.2 ± 0.28	385 ± 13.8	6.61 ± 0.32
**20**	**3.5**	0.0424 ± 0.00	0.240 ± 0.02	0.283 ± 0.02	0.179 ± 0.00	113 ± 3.93	409 ± 12.3	5.91 ± 0.10
	**0.6**	0.0363 ± 0.00 *	0.214 ± 0.02	0.250 ± 0.02	0.174 ± 0.01	102 ± 5.52	414 ± 9.85	5.88 ± 0.07
**25**	**3.5**	0.124 ± 0.00	1.07 ± 0.02	1.19 ± 0.02	0.117 ± 0.00	423 ± 6.88	356 ± 6.89	5.30 ± 0.11
	**0.6**	0.133 ± 0.00	0.94 ± 0.04	1.07 ± 0.04	0.143 ± 0.00 *	389 ± 6.00	367 ± 10.8	5.48 ± 0.09
**30**	**3.5**	0.233 ± 0.00	3.02 ± 0.02	3.25 ± 0.01	0.0774 ± 0.00	984 ± 12.9	302 ± 3.83	5.12 ± 0.11
	**0.6**	0.284 ± 0.00	2.84 ± 0.05	3.13 ± 0.05	0.1001 ± 0.00 *	982 ± 15.3	314 ± 2.89	4.98 ± 0.03
**35**	**3.5**	0.310 ± 0.02	6.06 ± 0.05	6.37 ± 0.03	0.0514 ± 0.00	1857 ± 35.5	291 ± 4.53	4.58 ± 0.03
	**0.6**	0.386 ± 0.01 *	5.69 ± 0.06 *	6.07 ± 0.06	0.0680 ± 0.00 *	1698 ± 40.0 *	279 ± 4.04	4.86 ± 0.05

^#^Percentage of dry biomass of the total fresh weight.

**Table A4:** Model selection results for the effects of NO_3_ supply on lettuce plant shoot dry mass, showing the top ranked models with a ΔAIC<10. Cross indicates that the fixed effect was included in the model.

Model rank	Run	[NO_3_]	[NO_3_]^2	Run:[NO_3_]	Run:[NO_3_]^2	AIC	ΔAIC	Loglik
1	+	+	+	+	+	−93.9	0.00	55.89
2	+	+	+	+		−93.3	0.61	54.36
3	+	+	+		+	−90.8	3.06	53.13
4	+	+	+			−85.1	8.76	49.09

**Table A5:** Parameter estimates from the top-ranked linear mixed effects model of lettuce shoot dry mass in response to nutrient solution N concentrations. Solution N concentration was log transformed (adding 0.1) in the analysis. Standard deviations (SD) and number of groups (n) are given for random effects on the intercept. 85 plants nested in 6 (Run 1) and 8 blocks (Run 2) were measured.

Shoot Dry Mass (g)		
Fixed effects:	β±SE	*p*-value
Intercept	1.23±0.043	<0.001
Run 1	0.45±016	0.005
N concentration	0.17±0.001	<0.001
N concentration ^2	−0.043±0.005	<0.001
Run 1: N conc.	0.35±0.15	0.023
Run 1: N conc.^2	−006±0.034	0.009
Random effects:	SD	n
Block	0.010	14
Residual	0.11	

**Table A6:** Parameter estimates from the top-ranked linear mixed effects models of N amount in plant tissues in response to solution N concentrations. Solution N concentration was log transformed (adding 0.1) in the analysis. Standard deviations (SD) and number of groups (n) are given for random effects on the intercept. 85 plants nested in 6 (Run 1) and 8 blocks (Run 2) were measured.

	N Shoot (g)		N Root (g)	
Fixed effects:	β ± SE	*p*-value	β ± SE	*p*-value
Intercept	39.79 ± 0.66	<0.001	45.10 ± 0.69	<0.001
Run 1	17.69 ± 3.17	<0.001	3.93 ± 3.81	0.305
N concentration	8.18 ± 0.22	<0.001	9.74 ± 0.27	<0.001
N concentration ∧2	−1.50 ± 0.097	<0.001	−1.81 ± 0.12	<0.001
Run 1: N conc.	−18.43 ± 3.15	<0.001	−7.79 ± 3.83	0.046
Run 1 : N conc. ^2	4.16 ± 0.71	<0.001	2.053 ± 0.87	0.021
Random effects:	SD	n	SD	n
Block	1.156	14	0.823	14
Residual	2.367		2.874	
