# Supplementary material for: Testing New Concepts for Crop Cultivation in Space: Effects of Rooting Volume and Nitrogen Availability
Source: Life (Basel). 2018 Oct 6;8(4):45. doi: 10.3390/life8040045 (PMC6316757; doi:10.3390/life8040045)
Supplement: Supplementary file 1 [file life-08-00045-s001.pdf]

Figure S1. Effects of nutrient solution volume over time

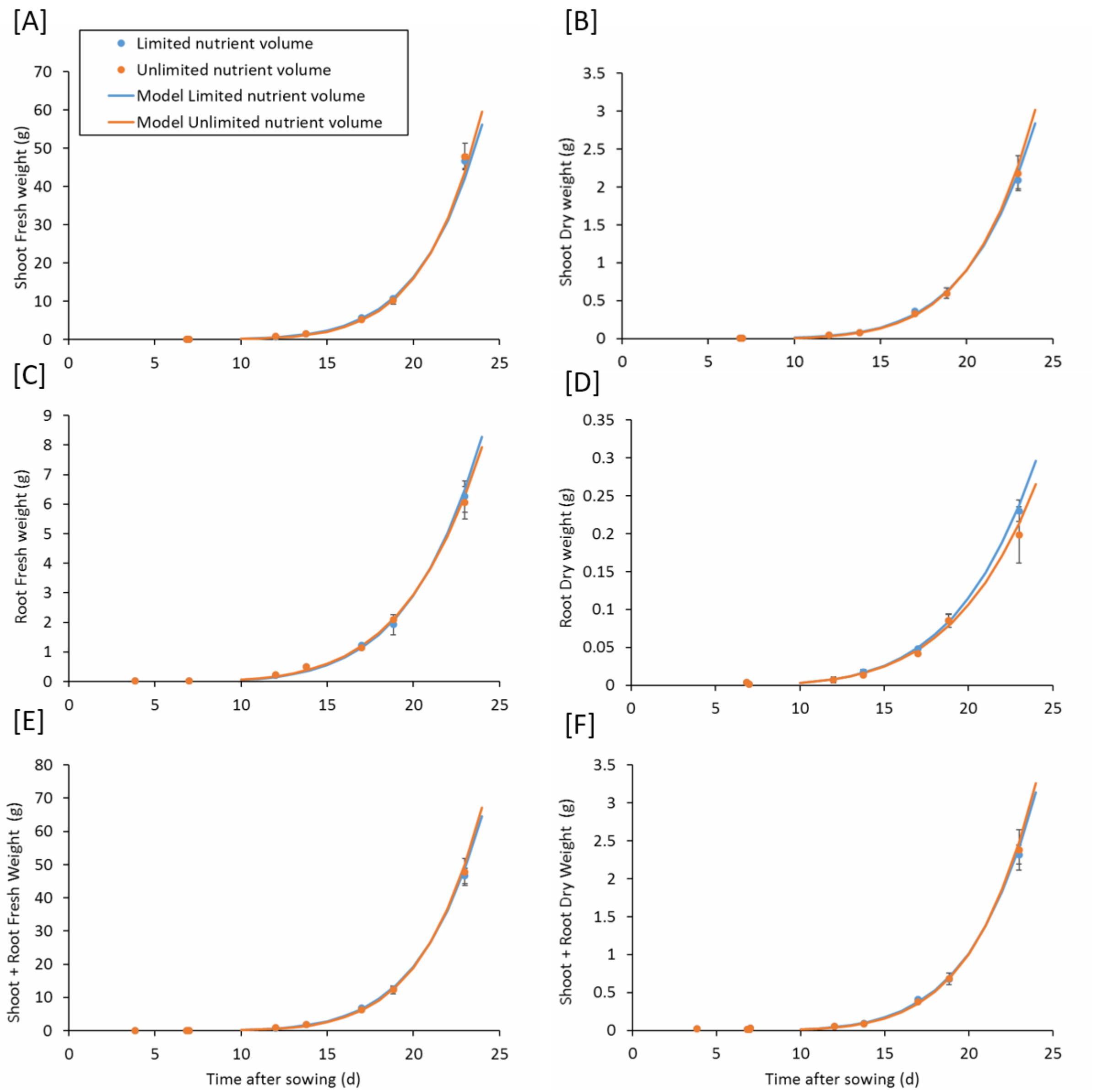

Figure S1.1: Plant weight (g) accumulation over time (d) for [A] fresh shoots, [B] dry shoots, [C] fresh roots, [D] dry roots, [E] whole fresh plants, and [F] whole dry plants. Error bars represent the standard error that was calculated for each harvest date. Lines represent the fitted growth rate using the model: biomass(time) = a*time^{b}. ($r^2 > 0.99$ for all models, see "Statistics" below for parameters values of each model.) Treatment means for each harvest were not significantly different as established by Tukey's Honestly Significant Difference test ($p < 0.05$).

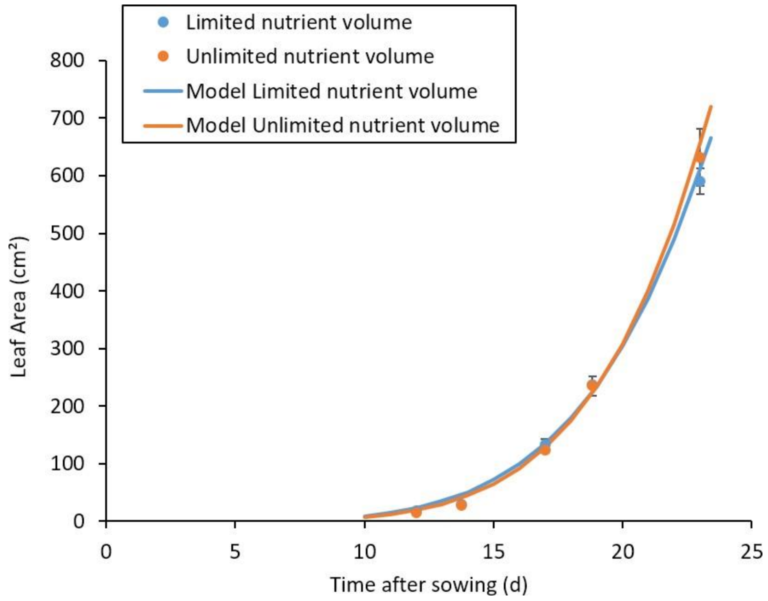

Figure S1.2: Leaf area (cm^2) over time (d) . Error bars represent the standard error that was calculated for each harvest date (n=2-6). Lines represent the fitted leaf area increase using the most parsimonious model: Leaf area(time) = a*time^b. (r^2 > 0.99 for both models, see 0 for parameters values of each model). Treatment means for each harvest were not significantly different as established by Tukey's Honestly Significant Difference test ($p < 0.05$).

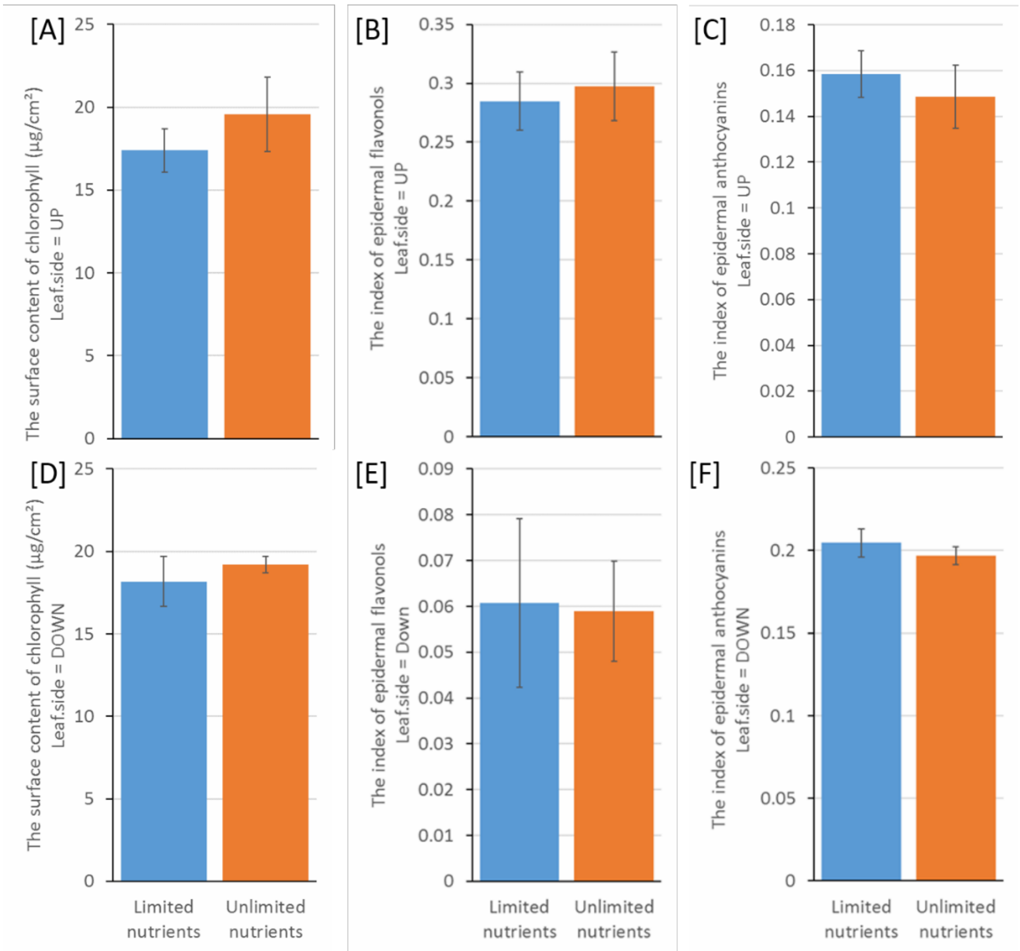

Figure S1.3: Dualex measurements of the upper side of the leaf of [A] the chlorophyll surface content, [B] index of epidermal flavonoids and [C] index of anthocyanins. [D – E] are respectively the same measures for the down side of the leaf. At harvest (day 24) the up and down side of 5 leaves of in total 6 plant per treatment were measured. None of the treatment means were significantly different as established by Tukey's Honestly Significant Difference test ($p < 0.05$).

Statistics: Model parameters of Figure S1.1 of biomass accumulation over time

This is a copy of the statistical output of R for the models that were fitted to the data presented in Sx. Formula: y ~ a * x^b

----
Whole plant fresh weight (FW) Unlimited nutrient solution

Parameters:
   Estimate Std. Error t value Pr(>|t|)
a 1.653e-08 1.989e-08   0.831   0.413
b 6.962e+00 3.851e-01 18.078 <2e^{-1}6 ***
Residual standard error: 1.674 on 29 degrees of freedom. R-square:[1] 0.9962016

Whole plant FW Limited nutrient solution

Parameters:
   Estimate Std. Error t value Pr(>|t|)
a 4.366e-08 3.047e-08   1.433   0.163
b 6.644e+00 2.235e-01 29.724 <2e^{-1}6 ***
Residual standard error: 1.023 on 27 degrees of freedom. R-square:[1] 0.9985692

Shoot FW Unlimited nutrient solution

Parameters:
   Estimate Std. Error t value Pr(>|t|)
a 6.184e-09 9.211e-09   0.671   0.509
b 7.233e+00 4.768e-01 15.171 8.65e^{-1}3 ***
Residual standard error: 1.695 on 21 degrees of freedom. R-square:[1] 0.9958449

Shoot FW Limited nutrient solution

Parameters:
   Estimate Std. Error t value Pr(>|t|)
a 2.350e-08 1.833e-08   1.282   0.214
b 6.795e+00 2.497e-01 27.214 <2e^{-1}6 ***
Residual standard error: 0.9531 on 21 degrees of freedom. R-square:[1] 0.9985776

Root FW Unlimited nutrient solution

Parameters:
   Estimate Std. Error t value Pr(>|t|)
a 2.192e-07 2.372e-07   0.924   0.366

b 5.476e+00 3.471e-01 15.775 4.06e^{-1}3 ***

Residual standard error: 0.2799 on 21 degrees of freedom. R-square:[1] 0.9944461

Root FW Limited nutrient solution

Parameters:
Estimate Std. Error t value Pr(>|t|)
a 9.686e-08 1.118e-07 0.866 0.396
b 5.747e+00 3.702e-01 15.523 5.54e^{-1}3 ***
Residual standard error: 0.2865 on 21 degrees of freedom. R-square:[1] 0.9945791

Whole plant dry weight (DW) Unlimited nutrient solution

Parameters:
Estimate Std. Error t value Pr(>|t|)
a 4.405e-09 6.332e-09 0.696 0.492
b 6.426e+00 4.605e-01 13.954 2.14e^{-1}4 ***
Residual standard error: 0.1137 on 29 degrees of freedom. R-square:[1] 0.9929137

Whole plant DW Limited nutrient solution

Parameters:
Estimate Std. Error t value Pr(>|t|)
a 9.736e-09 6.536e-09 1.49 0.148
b 6.164e+00 2.151e-01 28.65 <2e^{-1}6 ***
Residual standard error: 0.05529 on 27 degrees of freedom. R-square:[1] 0.9983047

Shoot DW Unlimited nutrient solution

Parameters:
Estimate Std. Error t value Pr(>|t|)
a 2.249e-09 3.775e-09 0.596 0.558
b 6.613e+00 5.377e-01 12.300 4.61e^{-1}1 ***
Residual standard error: 0.1161 on 21 degrees of freedom. R-square:[1] 0.9927955

Shoot DW Limited nutrient solution

Parameters:
Estimate Std. Error t value Pr(>|t|)
a 5.904e-09 4.636e-09 1.274 0.217
b 6.290e+00 2.516e-01 25.006 <2e^{-1}6 ***

Residual standard error: 0.05637 on 21 degrees of freedom. R-square:[1] 0.9981277

Root DW Unlimited nutrient solution

Parameters:

Estimate Std. Error t value Pr(>|t|)

a 3.093e-08 3.462e-08 0.893 0.382

b 5.023e+00 3.594e-01 13.977 4.16e^{-1}2 ***

Residual standard error: 0.01125 on 21 degrees of freedom. R-square:[1] 0.9922663

Root DW Limited nutrient solution

Parameters:

Estimate Std. Error t value Pr(>|t|)

a 2.053e-08 1.518e-08 1.352 0.191

b 5.187e+00 2.373e-01 21.858 6.3e^{-1}6 ***

Residual standard error: 0.007874 on 21 degrees of freedom. R-square:[1] 0.9969438

Leaf Aera (cm^2) Unlimited nutrient solution

Parameters:

Estimate Std. Error t value Pr(>|t|)

a 2.819e-05 3.427e-05 0.823 0.427

b 5.410e+00 3.899e-01 13.873 9.46e-09 ***

Residual standard error: 33.45 on 12 degrees of freedom. R-square:[1] 0.9935905

Leaf Aera (cm^2) Limited nutrient solution

Parameters:

Estimate Std. Error t value Pr(>|t|)

a 9.317e-05 5.698e-05 1.635 0.128

b 5.006e+00 1.963e-01 25.497 8.06e^{-1}2 ***

Figure S2. Electronic conductivity and pH in nutrient solution volumeexperiment

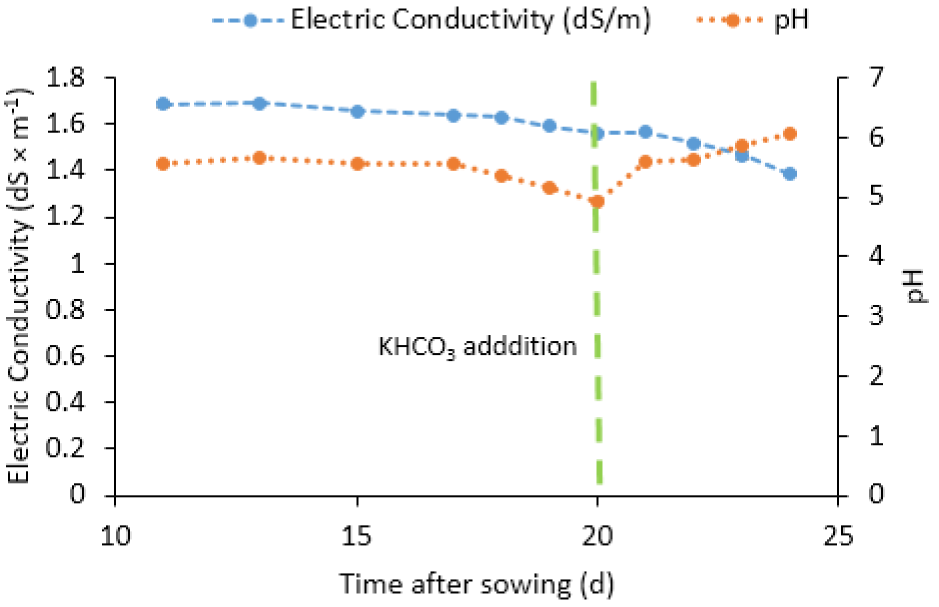

Electronic conductivity and pH over time (n=5) error bars fall within symbols. Note that on day 20, 2.4 ml of 0.1 mol·L^{-1} $KHCO_3$ was added to raise the pH to 5.6.

Figure S3. Ion concentration in nutrient solution volume experiment

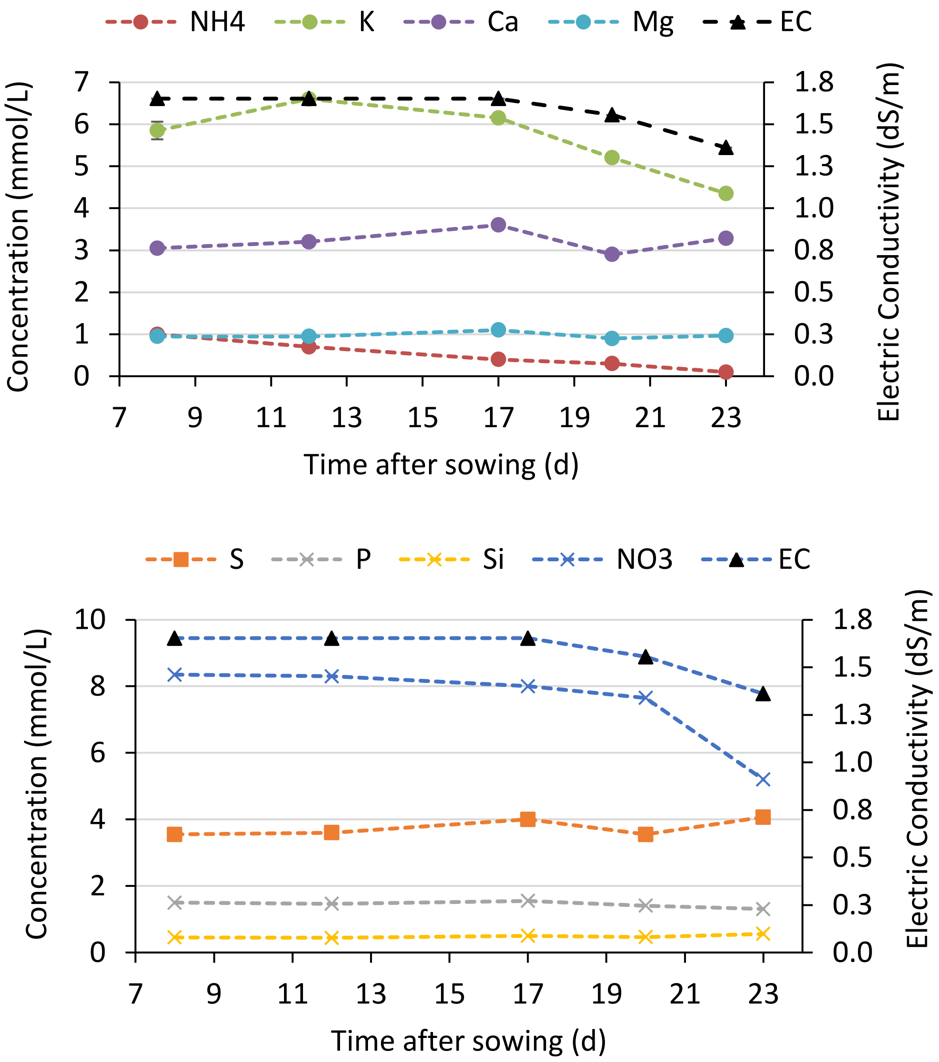

S4. Rosette size over time in relation to shoot compartment

The lettuce plants did not grow in a perfect symmetrical rosette and therefore the "smallest" and "largest" rosette radius can be determined. These rosette diameter measurements over time can be used to predict when the plant will start touching the walls of the crop cultivation chamber and when the plant is in full contact with the chamber or a neighbour plant (Figure S2). From these results it is clear that around 23 DAS the plants are in full contact with the crop cultivation chamber.

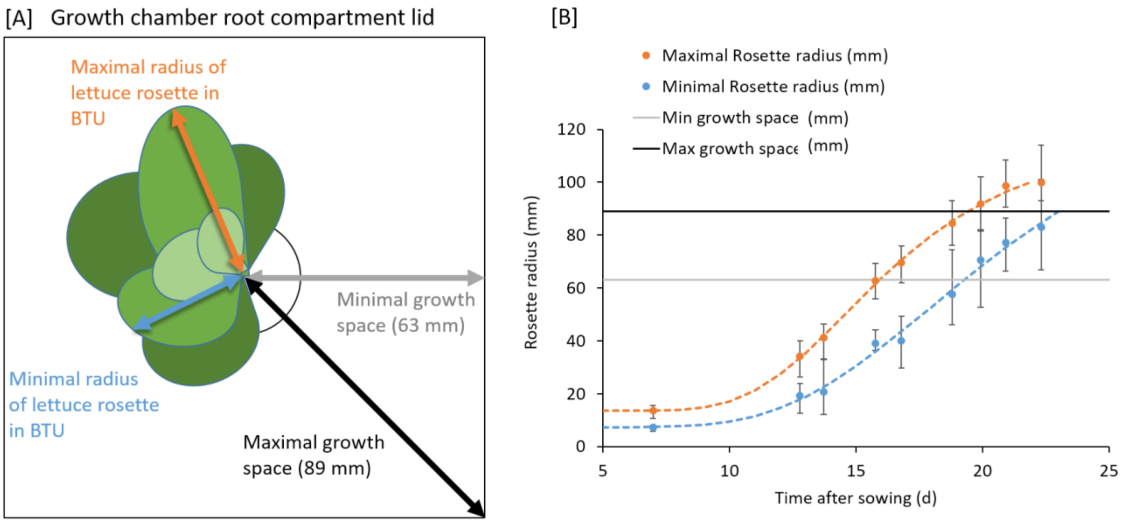

Figure S5. [A] Schematic representation of the minimal (blue) and maximal (orange) rosette radius of the lower leaves of a lettuce head on the root compartment lid of the crop cultivation system. The grey and black arrow show the minimal and maximal distance to the growth chamber wall (or a neighbour plant) respectively. [B] Mean minimal and maximal rosette radius over time (d). Bars represent the largest and smallest data points of the minimal and maximal rosette radius (n=8).

Lines represent the fit of an adjusted Gompertz cuve:

Rosette radius $(time)=A*\exp\left[-\exp\left(\frac{M*e}{A*(L-time)}+1\right)\right]+T$ Rosette radius $(time)=A*\exp\left[-\exp\left(\frac{M*e}{A*(L-time)}+1\right)\right]+T$

to the minimal ($r2 > 0.96$) and maximal ($r2 > 0.98$) rosette radius, were 'A' is the asymptote, 'T' the rosette radius at transfer, 'M' the maximum increase in rosette radius and 'L' the lag time to linear increase, . $r2 > 0.96$ (see table S4.1 for parameter values)

Model of MAX Rosette Radius (mm)

Formula: y ~ A * exp(-exp(M * exp(1)/A * (L - x) + 1)) + T

Parameters:

Estimate Std. Error t value Pr(>|t|)

A 100.1981 4.4909 22.31 <2e^{-1}6 ***

M 9.6677 0.5654 17.10 <2e^{-1}6 ***

L 10.7942 0.2556 42.23 <2e^{-1}6 ***

Signif. codes: 0 '***' 0.001 '**' 0.01 '*' 0.05 '.' 0.1 ' ' 1 Residual standard

error: 5.148 on 66 degrees of freedom

Number of iterations to convergence: 4

Achieved convergence tolerance: 4.208e-07 R-

square:[1] 0.9836755

Model of MIN Rosette Radius (mm)

Formula: y ~ A * exp(-exp(M * exp(1)/A * (L - x) + 1)) + T

Parameters:
Estimate Std. Error t value Pr(>|t|)
A 123.3738 24.6113 5.013 4.28e-06 ***
M 7.9296 0.3938 20.134 < 2e^{-1}6 ***
L 12.2287 0.3319 36.847 < 2e^{-1}6 ***

Signif. codes: 0 '***' 0.001 '**' 0.01 '*' 0.05 '.' 0.1 ' ' 1 Residual standard

error: 7.443 on 66 degrees of freedom Number of iterations to

convergence: 7

Achieved convergence tolerance: 2.753e-06
